# Immunopathogenesis of Emerging *Candida auris* and *Candida haemulonii* Strains

**DOI:** 10.3390/jof7090725

**Published:** 2021-09-05

**Authors:** Sujiraphong Pharkjaksu, Nawarat Boonmee, Chalermchai Mitrpant, Popchai Ngamskulrungroj

**Affiliations:** 1 Department of Microbiology, Faculty of Medicine Siriraj Hospital, Mahidol University, 10700 Bangkok, Thailand; sujiraphong.pha@mahidol.edu; 2 Department of Biochemistry, Faculty of Medicine Siriraj Hospital, Mahidol University, 10700 Bangkok, Thailand; nawarat.boo@mahidol.ac.th (N.B.); chalermchai.mit@mahidol.edu (C.M.)

**Keywords:** emerging *Candida*, innate immune response, multidrug resistance, zebrafish

## Abstract

The emergence of a multidrug-resistant *Candida* species, *C. auris* and *C. haemulonii*, has been reported worldwide. In Thailand, information on them is limited. We collected clinical isolates from Thai patients with invasive candidiasis. Both species were compared with a laboratory *C. albicans* strain. In vitro antifungal susceptibility and thermotolerance, and pathogenesis in the zebrafish model of infection were investigated. Both species demonstrated high minimal inhibitory concentrations to fluconazole and amphotericin B. Only *C. auris* tolerated high temperatures, like *C. albicans*. In a zebrafish swim-bladder-inoculation model, the *C. auris*-infected group had the highest mortality rate and infectivity, suggesting the highest virulence. The case fatality rates of *C. auris*, *C. haemulonii,* and *C. albicans* were 100%, 83.33%, and 51.52%, respectively. Further immunological studies revealed that both emerging *Candida* species stimulated genes involved in the proinflammatory cytokine group. Interestingly, the genes relating to leukocyte recruitment were downregulated only for *C. auris* infections. Almost all immune response genes to *C. auris* had a peak response at an early infection time, which contrasted with *C. haemulonii*. In conclusion, both emerging species were virulent in a zebrafish model of infection and could activate the inflammatory pathway. This study serves as a stepping stone for further pathogenesis studies of these important emerging species.

## 1. Introduction

The *Candida* species are important causes of bloodstream infections in hospitalized patients. This is especially the case in intensive care units, where patients receive broad-spectrum antimicrobial drugs, indwelling vascular catheters, parenteral nutrition, abdominal surgery, and immunosuppressive agents [1,2]. High mortality rates among patients have been associated with a delayed initiation of appropriate antifungal treatment [3,4]. This problem is compounded by the drug-resistant *Candida*, notably *C. glabrata*, found in many hospitals [5,6].

An emerging multidrug-resistant *Candida*, *C. auris*, was first reported in 2009 as an isolate from the external ear at a hospital in Japan [7]. In 2011, it was found in a bloodstream infection in Korea [8]. During the last decade, approximately 39 countries in East Asia, the Middle East, Africa, North America, South America, and Europe reported cases of *C. auris* infection [9]. *C. auris* might be resistant to multiple classes of antifungal agents, such as echinocandins and azoles. Moreover, it has the potential for person-to-person transmission [10]. Conventional microbiological methods often misidentify *C. auris* as *C. haemulonii*, a phylogenetically related drug-resistant *Candida* species that is also being increasingly reported in hospitals worldwide [11]. The first clinical isolation of *C. haemulonii* was collected from the hemoculture of a patient with renal failure which was reported by Lavarde et al. in 1984 [12]. A few years ago, treatment failures for *C. haemulonii* infections were associated with unresponsiveness to amphotericin B; reduced susceptibility to azoles and echinocandins has also been reported [13,14,15].

Considering the importance of these emerging human pathogens, it is imperative to understand the host defense mechanisms. However, the mechanisms regarding *C. auris* and *C. haemulonii* responses after infection are largely unknown [16]. Host defense against *Candida* species relies on an interaction between the innate and adaptive immune responses. Firstly, there is a physical barrier, consisting of the skin and mucosa. The second barrier is recognition of the *Candida* species by innate immune cells, such as neutrophils, monocytes, and macrophages. The recognition is driven by fungal pathogen-associated molecular patterns, which are mainly associated with fungal cell walls such as β-glucan or phospholipomannan (PLM) [17,18]. The subsequent release of proinflammatory cytokines, combined with the antigen-presentation activity of myeloid cells, is crucial for shaping the adaptive immunity, which represents a long-term barrier against fungal infection [19]. Recent studies showed that *C. auris* infection led to reduced neutrophil activities and macrophage lysis capacity [20,21]. In comparison to *Candida albicans*, a stronger cytokine response was also observed in *C. auris* which mediated through the recognition of C-type lectin receptors. Collectively, *C. auris* was less virulent than *C. albicans* in in vivo experimental models of disseminated candidiasis [21]. However, studies of host immune response to *C. haemulonii* was still lacking.

Some studies on bacteria have reported finding an association between the antibiotic resistance and host pathogenesis [22,23]. In our study, we investigated the interaction between emerging *Candida* strains, *C. auris* and *C. haemulonii*, exhibiting high MICs to antifungal agents and host immune response by using zebrafish as a model for an immunopathogenesis study. Upon finishing this work, we found that *C. auris* and *C. haemulonii* were both highly virulent and several proinflammatory cytokine genes were involved in their pathogenesis. This highlights the advantage of using the zebrafish model to determine the virulence of *Candida* species, and potentially to investigate these emerging drug-resistant *Candida* species in the future.

## 2. Materials and Methods

### 2.1. Clinical Isolates

*C. auris* and *C. haemulonii* isolates were collected from the yeast culture collection held by the Mycology Unit, Department of Microbiology, Faculty of Medicine Siriraj Hospital, Mahidol University, Thailand. The isolates had been obtained from invasive candidiasis patients at the hospital, firstly in 2018 (from blood tested for the *C. auris* strain SI-18-CAU-HEM) and later in 2021 (from pleural effusion tested for the *C. haemulonii* strain SI-21-CH-PLF).

Species identification was confirmed by ITS sequencing (ATCG Company Ltd., Thailand). The ITS sequences were compared to reference sequences deposited in the GenBank Databases (https://blast.ncbi.nlm.nih.gov accessed on 28 May 2021). Accurate species identification targets included an *E*-value of ≤ 10 ^5^ and identity and coverage of ≥98% [24].

The nucleotide sequences of *C. auris* and *C. haemulonii* were assigned NCBI database accession numbers MZ312603 and MZ312604, respectively, and the ITS phylogenetic tree was represented in Figure S1. Before commencement of this research, its protocol was approved by the ethics committee of the Siriraj Institutional Review Board (Si. 091/2016; 9 February 2021).

### 2.2. Thermotolerance and Antifungal Susceptibility Testing

Assessment of thermotolerance was performed by spotting serial dilutions of *C. auris* and *C. haemulonii*, plus the control strain, *C. albicans* ATCC24433, on Sabouraud dextrose agar (SDA) plates and assessing growth after 48 h incubation at 30 °C, 37 °C, and 42 °C.

The susceptibility of the yeast strains to antifungal drugs was determined by using Sensititre YeastOne YO10 (SYO; Thermo Fisher Scientific, Waltham, MA, USA), a colorimetric microdilution method, as per the manufacturer’s instructions. Nine drugs were used: fluconazole, voriconazole, itraconazole, posaconazole, 5-flucytosine, anidulafungin, micafungin, caspofungin, and amphotericin B.

### 2.3. Zebrafish Maintenance and Infection Experiment

Adult wild-type zebrafish (*Danio rerio*, *Tuebingen/AB* strain) were kept in recirculated water aquarium under an alternated light/dark cycle of 14 h and 10 h, respectively. Larval zebrafish were incubated at 28.5 °C in E3 buffer (60× stock solution: 34.8 g NaCl, 1.6 g KCl, 5.8 g CaCl_2_·2H_2_O, 9.78 g MgCl_2_·6H_2_O with 100 µL of 1% methylene blue [MB] in working solution). For the infection experiments, the larvae were manually dechorionated between 24- and 30-hour post-fertilization. Prior to microinjection, the larvae were anesthetized in E3-MB containing 0.2 mg/mL tricaine (ethyl-3-aminobenzoate; Sigma-Aldrich) [25].

The yeast strains were cultured in yeast-peptone-dextrose agar (YPD; Becton Dickinson, Franklin Lakes, NJ, USA). The plated cultures were inoculated to 5 mL liquid YPD and grown for 18 h at 30 °C in a shaking incubator at 250 rpm. Yeast cells were centrifuged at 8000× *g*, washed twice with phosphate buffer saline (PBS), and resuspended in 2 mL PBS. The concentration of the yeast suspension was adjusted by counting under a light microscope with a hemacytometer (INCYTO C-CHIP, Korea). This diluted suspension was pelleted at 8000× *g* for 15 min and resuspended in autoclaved 10% polyvinylpyrrolidone 40 (PVP40) with 0.5% phenol red in PBS [26].

The yeast cells were inoculated to zebrafish larvae 5 days post-fertilization (dpf) using a microinjector. Three nanoliters of the yeast suspension (adjusted to 150 and 250 yeast cells) were injected into the swim bladders of the zebrafish larvae [27]. Mock injected control and infected zebrafish larvae were kept in E3 buffer at 30 °C. This study was approved by the Siriraj Animal Care and Use Committee (SiACUC) (020/2562; 27 January 2021).

### 2.4. Survival Rate

The survival study was performed using 26 larvae per group. Survival of infected zebrafish larvae was recorded daily up to day nine.

### 2.5. Infectivity Assessment

Infectivity was determined by colony forming unit (CFU) quantification in biological triplications, based on minor adaptation of a previous study [28]. Five representative infected larvae were pooled and homogenized at 8 hpi (hour-post-infection), 24 hpi, and 96 hpi in 50 μL of 1X PBS. For plating, 20 μL of homogenate from each group were plated on YPD agar supplemented with antibiotics (penicillin/streptomycin). To achieve a countable number of colonies, homogenate (undiluted sample), 1:10, 1:100, 1:1000, and 1:10000 dilutions were plated for sample at each time point. Plates were incubated overnight at 30 °C, and colonies were counted the following day. Biological triplication was undertaken to ensure consistency of the experimental results.

### 2.6. RNA Extraction and Expression Analysis

RNA was isolated from 8 and 96 hpi larvae by using RNeasy Mini Kit (Qiagen, Hilden, Germany) following the manufacturer’s instructions. To synthesize cDNA, total of 50 ng of RNA were used in a reverse transcription reaction by using iScript Reverse Transcriptase (Bio-Rad, USA).

Each 20 µL reaction mixture of real time PCR contained 5 µL of 40 ng cDNA, 50 nM concentrations of each gene-specific primer (Table S1), and 10 µL of LightCycler 480 SYBR Green I Master Mix (Roche Life Science, Penzberg, Germany). Real-time PCR was performed on a Roche LightCycler 480 machine. cDNA quantitation was performed in triplicates, and reactions were normalized against the β-actin gene as an internal control [22]. cDNA was amplified with an initial denaturation at 95 °C for 10 min before 40 cycles of denaturation (95 °C for 10 s), annealing (52–58 °C for 20 s), and extension (72 °C for 20 s). This was then followed by a melting curve and cooling step. Determination of expression was calculated by normalized expression ratio (2^−∆∆CT^) compared with the β-actin gene.

### 2.7. Statistical Analysis

Statistical analyses were performed using GraphPad Prism 8. In vivo in zebrafish data were assessed with the Mantel–Cox test to determine survival analysis. CFU quantifications and normalized gene expressions at different timepoints were compared by ordinary one-way ANOVA with Tukey’s multiple comparison test. In all cases, *p* < 0.05 was deemed significant.

## 3. Results

### 3.1. Antifungal Susceptibility and Thermotolerant Testing

The MICs of the *C. auris* and *C. haemulonii* strains investigated in this study are presented in Table 1. Compared with *C. albicans* ATCC24433, these emerging *Candida* strains showed high MICs for fluconazole: the MIC of *C. auris* was 512 times higher, while that of *C. haemulonii* was 64 times greater than the *C. albicans* strain. Moreover, the *C. auris* strain had high MICs for other azoles and amphotericin B.

**Figure 1:**
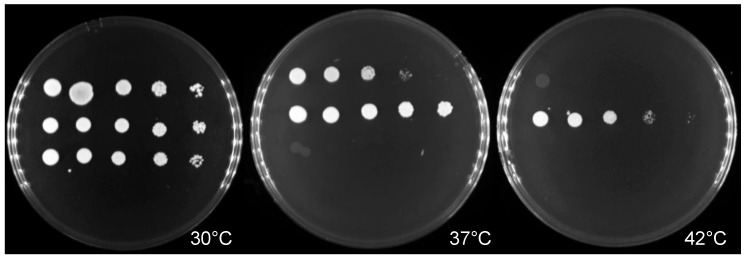
Differing thermotolerances of *Candida albicans, C. auris,* and *C. haemulonii*. Sabouraud dextrose agar plates showing growth of representative *Candida* strains after 48 h incubation at 30–42 °C with serial dilution spots. Top row: *C. albicans* ATCC24433 as the control strain; middle row: *C. auris* strain SI-18-CAU-HEM; and bottom row: *C. haemulonii* strain SI-21-CH-PLF.

Growth at a physiological temperature is a prerequisite for microbial invasion and pathogenicity. The *C. haemulonii* isolates grew well at 30 °C, but their growth was poor or absent at 37 °C, and no growth occurred at 42 °C. In contrast, while the *C. auris* and *C. albicans* isolates were able to grow in temperatures ranging from 30 °C to 42 °C, *C. auris* demonstrated better growth than *C. albicans* (Figure 1).

### 3.2. Survival in a Zebrafish Model of a Mucosal Candida Infection at the Swim Bladder

**Figure 2:**
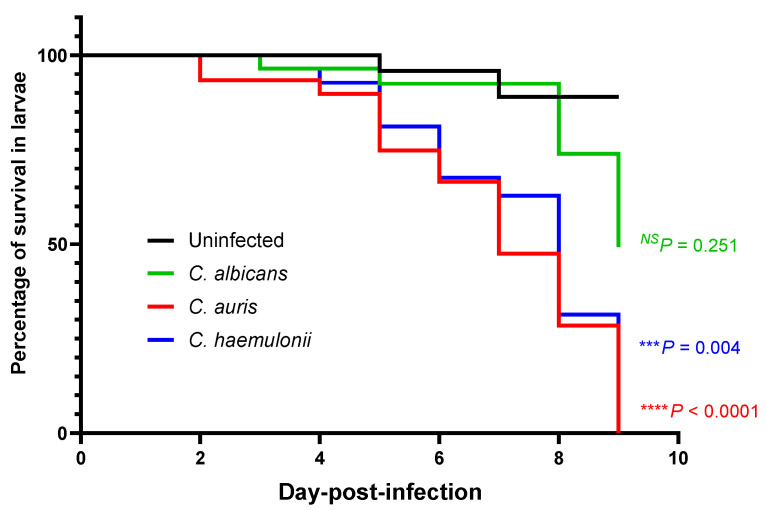
Emerging *Candida* virulence in a zebrafish model. Each experimental group of 26 zebrafish was injected with 10^7^ CFU cells of one of the *C. albicans* ATCC24433 (control) strain, *C. auris* strain SI-18-CAU-HEM, or *C. haemulonii* strain SI-21-CH-PLF. Each experiment was performed in duplicate.

To evaluate the virulences of the *C. albicans, C. auris*, and *C. haemulonii* strains in a zebrafish model of a mucosal *Candida* infection at the swim bladder, their survival was monitored over the course of nine days after infection (Figure 2). Although inoculation with the *C. albicans* strain resulted in 50% zebrafish death but the difference was not significant compared to the control group with 10% zebrafish death (*p* = 0.251). Interestingly, inoculation with *C. auris* produced 100% zebrafish death (*p* < 0.0001), whereas *C. haemulonii* produced 80% zebrafish death (*p* = 0.004) when comparted to the death in the control group. This result indicates that the *C. auris* and *C. haemulonii* strains had more virulence than the *C. albicans* strain.

### 3.3. Fungal Burden in Candidiasis Zebrafish Model

**Figure 3:**
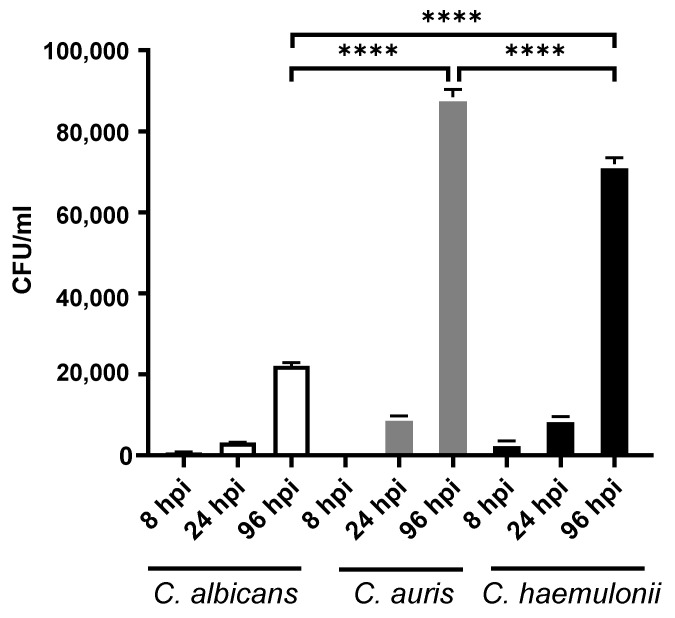
Fungal burden in zebrafish model. Each experimental group of five zebrafish was injected with 10^7^ cells of one of the *C. albicans* ATCC24433 (control) strain, *C. auris* strain SI-18-CAU-HEM, and *C. haemulonii* strain SI-21-CH-PLF. Each experiment was performed in triplicate. Abbreviations and symbols: CFU/mL, colony forming unit per milliliter; ****, *p* < 0.0001.

The ability to colonize within a host is essential for *Candida* infections. For this reason, a CFU was used to examine *Candida* within the zebrafish. As illustrated in Figure 3, the fungal burden in all groups of fish was counted at different timepoints. The number of *Candida* cells significantly increased from 24 to 96 hpi in all groups (*p* > 0.0001), which indicated that there was cell proliferation. In particular, at 96 hpi, the number of cells in fish injected with *C. auris* was the highest, followed by *C. haemulonii* and *C. albicans*. This correlated with the survival analysis results.

### 3.4. Dynamics of Immune Response Genes to Emerging Candida in Zebrafish Model

During an infection with *Candida*, the host immune system recognizes the pathogen-associated molecular patterns and induces the expression of cytokines [29]. To examine the pathogen-zebrafish interactions, the expression profiles of the host immune response genes that played an important role to activate immune cells and secrete cytokines or other components during *Candida* infection were monitored by real-time quantitative PCR (qPCR) at 8 hpi (as the early timepoint) and 96 hpi (as the late timepoint). The raw data of normalized gene expression was shown in Table S2 and Figure S2. The fold changes of the gene expression levels in zebrafish infected with *C. auris* and *C. haemulonii* were compared with the expression in zebrafish infected with the *C. albicans* control strain (Table 2).

**Figure 4:**
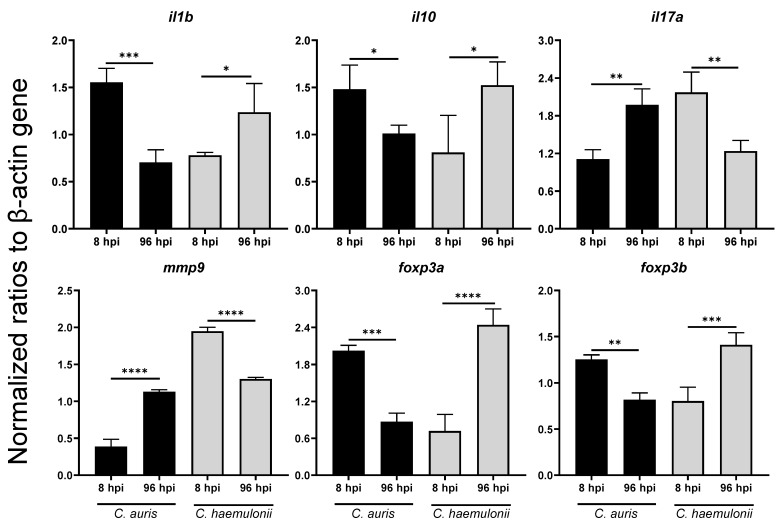
The expression levels of *il1b, il10, il17a, mmp9, foxp3a,* and *foxp3b* in zebrafish infected with emerging *Candida* strains, by timepoint. Each experimental group of 20 zebrafish was injected with 10^7^ CFU cells of one of the *C. auris* strain SI-18-CAU-HEM or the *C. haemulonii* strain SI-21-CH-PLF. The normalized ratios of expression were calculated by comparison with the level of expression of the β-actin gene in each group at 8 hpi. Each experiment was performed in triplicate. **Abbreviations and symbols:** hpi, hour-post-infection; *, *p* < 0.5; **, *p* < 0.01; ***, *p* < 0.001; ****, *p* < 0.0001.

Most of the proinflammatory and inflammatory cytokine expression genes were upregulated in the *C. auris* and *C. haemulonii* infection. Compared with the *C. albians* group, the normalized ratios of expression were at significantly higher levels for both the *C. auris* and *C. haemulonii* groups (for example, *tnfa*, *il1b*, *il8*, *il10*, and *il17a*). There was a high fold change of expression in *C. auris* group during the early timepoint, but the *C. haemulonii* group peaked at the late timepoint. Moreover, we found significant downregulation in *il6* in the *C. auris* infection at the late phase (Table 2 and Table S3, Figure 4). The expressions of *tnfa*, *il8*, and *il10* in the *C. auris* and *C. haemulonii* groups were at a higher level than in the *C. albicans* group at both the early and late timepoints (Figure S3).

To further understand the role of leukocytes and their activities to defend *Candida*, we determined the expression of *inos* (nitric oxide synthase in macrophage) and *mpx* (myeloperoxidase in leukocytes). No significant differences in the expression levels of either *inos* and *mpx* were detected among the three groups at the early timepoint. Interestingly, the *mpx* expression in the *C. auris* infection was significantly downregulated at the late timepoint. The results are illustrated in Table 2.

Next, the matrix metalloproteinase expressions as the leukocyte recruitment mediator [30] revealed that the matrix metalloproteinases genes were significantly upregulated in *C. auris* and *C. haemulonii* in both phases. Focusing on *mmp9*, the *C. auris* infected group had a significantly high expression at the late phase, whereas the *C. haemulonii* group surged at the early phase (Figure 4). As to the inflammatory regulatory gene expression, most genes were activated at a similar level to that of the *C. albicans* infection, with the *jak2* expression at a significantly increased level in *C. auris* at the early timepoint (Table 2). Interestingly, *nfkb* demonstrated higher fold change expressions after the *C. auris* and *C. haemulonii* infections than the *C. albicans* group (Figure S3).

Moreover, we detected the gene expressions of *foxp3a* and *foxp3b*, given their importance in the development and function of regulatory T-cells [31]. The results revealed that the *C. haemulonii* infection had expressions that were at a similar level to those of the *C. albicans* infection, while the *C. auris* infection showed a significantly high expression for both genes (Table 2). Additionally, we found a different timepoint of gene expression among the strains. The *C. auris* infection was significantly upregulated at the early timepoint, but the *C. haemulonii* infection increased at the late timepoint (Figure 4).

## 4. Discussion

The global emergence and spread of *C. auris* as a causative agent of invasive nosocomial infection has arisen from resistance to multiple antifungal drugs and possibly to all major classes of systemic antifungal drugs [5,32]. In addition, there has been horizontal transmission among hospitalized patients [32,33]. *C. auris* and *C. haemulonii* are phylogenetically related species in the *Metschnikowiaceae* family, and they have multidrug-resistance properties [34]. We studied the characteristics of the first-isolated *C. auris* and *C. haemulonii* at our hospital, and we highlighted experimental evidence to identify differences in their drug susceptibility patterns, pathogenicity, and host responses in zebrafish. *C. albicans* was used as the reference strain.

In our study, *C. auris* and *C. haemulonii* demonstrated high MICs for fluconazole and amphotericin B, while echinocandin MICs were within the susceptible range, according to the tentative MIC breakpoint value that had been previously established [13,35]. This result was in concordance with the findings of other studies, namely, that high MICs for triazoles and amphotericin B among emerging *C. auris* and *C. haemulonii* strains were increasingly apparent in clinical settings [11,35,36,37,38,39,40]. Thermotolerant testing revealed that *C. albicans* and *C. auris*—but not *C. haemulonii*—could grow at 30–42 °C, and that *C. auris* had the highest thermotolerance. This corresponds with the findings of recent publications [41,42]. Our survival analysis showed that *C. auris* exhibited the highest mortality rate of infection, followed by *C. haemulonii* and *C. albicans*. As to the fungal burden in the experimental model of zebrafish, the *C. auris* with the highest thermotolerance had the highest infectivity.

Based on our findings, *C. auris* was more virulent and caused earlier mortality of infected larvae. This is consistent with other work, which reported that this species demonstrated more severity than *C. haemulonii* in animal studies [43,44]. One United Kingdom study reported that *C. auris did* not form cellular aggregates, thereby causing a significant virulence in terms of the mortality rate [45]. Additionally, the genomes of *C. auris* and *C. haemulonii*, which are closely related species, contain *C. albicans* gene orthologs, such as proteinases and mannosyl transferases, which might play roles in pathogenesis. However, the genes of *C. albicans* have not been characterized [46]. However, previous studues reported that *C. auris* and *C. haemulonii* were less virulent than other *Candida* species (such as *C. albicans* and *C. tropicalis*) [41,47,48]. This might be explained by the fact that *C. auris* and *C. haemulonii* lacked the ability to produce hyphae, which is an important virulence factor for disseminated infections. Therefore, a comparison between swim bladder, performed in this study, and intravenous route of infections would be required and warranted in the next study. An interesting factor in our study was the high MICs for antifungals of the emerging *Candida* isolates. This factor might cause increasing severity or virulence from pathogens, as presented by other studies on *Staphylococcus aureus* [49], *Escherichai coli*, *Pseudomonas aeruginosa* [23], and *Vibrio alginolyticus* [50].

To understand the pathogenesis of emerging *Candida* strains, we performed gene expression analysis to observe the host immune response to pathogens in systemic infections. Firstly, proinflammatory cytokines play a role in stimulating immune cells (especially macrophages) to destroy pathogens [51]. The expression of most proinflammatory cytokine genes was upregulated at the early phase with *C. auris*. Although a similar pattern was observed with *C. haemulonii*, some genes (such as *il1b* and *il10*) peaked at the late phase. On the other hand, *Il10*, an inflammatory cytokine, was induced in an early phase of *C. auris* infection. This inflammatory cytokine could potentially be released by Toll like receptor 2 (TLR2) dependent pathway in macrophage and hampered proinflammatory cytokines that is critical for neutrophil recruitment [52,53]. Interestingly, *il17α* activation as a key to the cytokine gene that links to neutrophil recruitment [54] showed a slow response after *C. auris* infection and *il8* and *mpx*, surrogate markers for neutrophil function, were significantly reduced in later phase of *C. auris* infection. This finding contrasted with *C. albicans* and *C. haemulonii* infection in that their responses occurred at the early timepoint. This result suggests that the neutrophil function in a host infected with *C. auris* is less capable than that of neutrophils in larvae infected with *C. haemulonii* and *C. albicans* and may partly be an explanation for virulence of *C. auris*.

Matrix metalloproteinase (MMPs) are members of the proteolytic enzyme family and play multiple roles in the normal immune response to infection, including leucocyte recruitment, cytokine, and chemokine processing, and defensin activation [55]. We found that the expression of *mmp9* had an opposing pattern to proinflammatory cytokine genes such as *tnfa* and *il1b*, which supports the role of MMPs in controlling the production of proinflammatory cytokines [56]. The key to proinflammatory cytokine production is the activation of the transcription factor, NFkB, after the toll-like receptors (TLRs)–pathogen interaction in innate immunity. Almost all TLRs signal via MyD88 as an adaptor protein for NFkB activation, with subsequent inflammatory cytokine production and control of adaptative immunity [57,58]. The data correlated to the previous description as the myd88/nfkb gene expressions were stimulated by pathogen. The levels of expression fluctuated due to different modes of activation, demonstrating that NF-kB activation is an important requirement for the expression of many *Candida*-regulated genes [59]. As to other transductors, JAK/STAT is the signal transduction pathway of many essential cytokines involved in sepsis [60].

Lastly, regulatory T (T_reg_) cells play a major role in the suppression of excessive immune responses. The functions of these cells were controlled by the expression of regulatory gene encoding the forkhead box P3 (FOXP3) protein [61,62]. Our results showed that *Candida* infection (other than *C. auris*) activated *foxp3a* and *foxp3b* at the late timepoint suggesting that *C. auris* strain might reduce the number of regulatory T cells at the late time point and allow to increase yeast population compared to other *Candida* species. This data agreed with a previous study [63] that demonstrated the roles of *foxp3a* and *foxp3b* in suppressing inflammatory cytokine secretion and T cell maintenance in zebrafish. Moreover, that earlier research identified that *foxp3a* and *foxp3b* could stimulate the IL-17-secreted cell response to *Candida* infection; this corresponded with the findings of the current study [64].

As mentioned earlier, the effects of drug resistant isolate might be influenced by the host immune response. Jiang JH et al. [49] recorded that the daptomycin-resistant *Staphylococcus aureus* strain had impaired neutrophil recruitment in vivo and promoted bacterial survival. Moreover, gene expression of proinflammatory cytokines and molecules of innate immunity (such as lysozyme and C3b in zebrafish larvae infected with ceftazidime-resistant [50] and levofloxacin-resistant [65] *Vibrio alginolyticus* strains) showed higher upregulation than susceptible strains.

In summary, the strains of emerging *Candida* species, *C. auris*, and *C. haemulonii*, with high MICs for antifungal agents, showed significantly higher virulences than the *C. albicans* control strain used in the zebrafish model. In terms of the immune response, differences in the patterns of gene expression were noted, especially for *C. auris*. The benefits of using a zebrafish model to study the pathogenesis of fungal infections were considered. Our results highlight the potential of using zebrafish as an effective model for the investigation of the mechanisms controlling infections as well as for therapeutic efficiency studies. As the number of strains in this study was limited due to very few cases at our hospital, we need to collect more strains for future research.

## Figures and Tables

**Table 1:** Antifungal susceptibility test results of the *C. albicans* (control strain), *C. auris*, and *C. haemulonii* strains.

Strain	Minimum Inhibitory Concentration (MIC, ug/mL)								
	AND	MF	CAS	AB	5FC	PZ	VOR	IZ	FZ
*C. albicans* ATCC24433	≤0.015	≤0.008	0.015	0.12	≤0.06	0.03	0.015	≤0.015	0.5
*C. auris* SI-18-CAU-HEM	0.12	0.12	0.25	4	0.25	≥8	≥8	≥16	≥256
*C. haemulonii* SI-21-CH-PLF	0.12	0.25	0.12	2	0.5	0.25	0.25	0.5	32

Abbreviations: 5FC, 5-flucytosine; AB, amphotericin B; AND, anidulafungin; CAS, caspofungin; MF, micafungin; IZ, itraconazole; FZ, fluconazole; PZ, posaconazole; VOR, voriconazole.

**Table 2:** Fold changes of gene expression levels of zebrafish infected with *C. auris* and *C. haemulonii* at different timepoints, compared with *C. albicans* infection.

Genes	Early Timepoint (8 hpi)		Late Timepoint (96 hpi)	
	*C. auris*	*C. haemulonii*	*C. auris*	*C. haemulonii*
**Proinflammatory and Inflammatory Cytokines**				
*tnfa*	3.74 ****	2.60 *	2.13 *	2.06 *
*ifng*	1.22 *^NS^*	1.27 *^NS^*	0.92 *^NS^*	1.76 *^NS^*
*il1b*	2.43 ***	1.22 *^NS^*	1.12 *^NS^*	1.96 **
*il6*	0.97 *^NS^*	1.25 *^NS^*	0.63 **	1.05 *^NS^*
*il8*	4.72 ****	2.37 ***	2.05 *^NS^*	2.51 **
*il10*	2.47 **	1.35 *^NS^*	1.33 *^NS^*	2.00 **
*il17a*	1.18 *^NS^*	2.31 ***	1.25 *^NS^*	0.78 *^NS^*
**Leukocyte Activities**				
*inos*	1.07 *^NS^*	0.93 *^NS^*	0.96 *^NS^*	0.85 *^NS^*
*mpx*	0.98 *^NS^*	0.83 *^NS^*	0.42****	1.04 *^NS^*
**Matrix Metalloproteinases**				
*mmp9*	0.76 *^NS^*	3.82 ****	2.09 ****	2.41 ****
*mmp13*	1.62 *	1.72 *	1.74 *	1.88 **
**Inflammatory Regulators**				
*myd88*	1.54 *^NS^*	1.06 *^NS^*	0.92 *^NS^*	1.05 *^NS^*
*nfkb*	1.02 *^NS^*	1.56 **	1.17 *^NS^*	2.11 ****
*jak2*	3.04 ****	0.46 *	0.93 *^NS^*	0.52 *^NS^*
*stat3*	0.87 *^NS^*	1.17 *^NS^*	1.23 *^NS^*	1.53 *
**Regulatory T-Cells**				
*foxp3a*	3.16 ****	1.12 *^NS^*	0.33 ****	0.92 *^NS^*
*foxp3b*	1.49 **	0.96 *^NS^*	0.56 ****	0.97 *^NS^*

**Note:** The expression folds were analyzed with the Livak method (2^−∆∆CT^): <1, downregulation; 1, basal; >1, upregulation. **Abbreviations and symbols:** hpi, hour post infection; *^NS^*, not significant; *, *p* < 0.5; **, *p* < 0.01; ***, *p* < 0.001; ****, *p* < 0.0001.

## Data Availability

Not applicable.

## Supplementary Materials

The following are available online at https://www.mdpi.com/article/10.3390/jof7090725/s1. Table S1: Primers used for qPCR experiments in this study [22,28,63,66,67,68,69]; Table S2: Average of gene expression levels of zebrafish infected with *C. auris* and *C. haemulonii* at different timepoints, compared with the expression level of β-actin in each group; Figure S1: Phylogenic tree using ITS sequences of emerging strains in this study [70]; Figure S2: qPCR analysis of the gene expression levels in zebrafish infected with *Candida albicans* ATCC24433 as a control strain in this study; Figure S3: qPCR analysis indicating the high alterations of the expression levels of *tnfa, il8, il10,* and *nfkb* in zebrafish infected with emerging *Candida* strains.
