# Supplementary material for: Immunopathogenesis of Emerging Candida auris and Candida haemulonii Strains
Source: J Fungi (Basel). 2021 Sep 5;7(9):725. doi: 10.3390/jof7090725 (PMC8469599; doi:10.3390/jof7090725)
Supplement: Supplementary file 1 [file jof-07-00725-s001.zip › jof-1346291-supplementary.pdf]

Journal of Fungi

MDPI

Table S1. Primers used for qPCR experiments in this study.

Primer	Sequence (5′ – 3′)	Reference
B-actin_F	GCTGACAGGATGCAGAAGGA	[66]
B-actin_R	TAGAAGCATTTGCGGTGGAC	
IL1-B_F	TTGTGGGAGACAGACAGTGC	
IL1-B_R	GATTGGGGTTTGATGTGCTT	
TNF-a_F	ACAAGGCAATTTCACTTCCA	
TNF-a_R	AGCTGATGTGCAAAGACACC	
IL-10_F	TCACGTCATGAACGAGATCC	
IL-10_R	CACCATATCCCGCTTGAGTT	
iNOS_F	GAGCAGGCCCAATGCATTT	
iNOS_R	TGCGCTGCTGCCAGAAAC	
IFN-Y_F	CTATGGGCGATCAAGGAAAA	
IFN-Y_R	CTTTAGCCTGCCGTCTCTTG	
MyD88_F	TAAACGGCTAATCCCTGTCG	[67]
MyD88_R	TCGAGTCCAGAACCAGACCT	
IL-6_F	GGACGTGAAGACACTCAGAGACG	[28]
IL-6_R	AAGGTTTGAGGAGAGGAGTGCTG	
IL-17a_F	CAATCTGAGGACGGAAAGGG	
IL-17a_R	ACTGGGCTTCAAAGATGACC	
NF-kB P65F1	GGCAGGTGGCGATAGTGTT	[68]
NF-kB P65R1	CATTCCTTCAGTTCTCTTGCG	
jak2F1	TCGCTGCTTCTTCTGTCAGG	
jak2R1	ACAGCCGTCCATTTTGGCTT	
stat3F1	CATTCCCAAAGAGGAGGCGT	
stat3R1	ATTTGGCTCGGAGAGAAAGCA	
foxp3a_F	ACCTCACCAAAACCAATGGC	[63]
foxp3a_R	TCTGATTGCGAGATGAGCCG	
foxp3b_F	GCGTGGACGAGACTTTTTCT	
foxp3b_R	GCAGCTGAAGAAACTGCATTC	
mpx_F	TTCCAGAAAATCCGAGATGG	
mpx_R	CCATTGACACAGAAGCCAGA	
IL-8_F	TTTTCCTGGCATTTCTGACC	[22]
IL-8_R	CGTCGGCTTTCTGTTTCAAT	
mmp9_F	CCCTTGGCTCTGGACCAGCC	[69]
mmp9_R	CTGTGCGGCCCTCAGTGGTG	
mmp13_F	CCGCCTGCTGGAGGAGACCA	
mmp13_R	GACGGGCTCGGGTTCTGTGC	

Table S2. Average of gene expression levels of zebrafish infected with *C. auris* and *C. haemulonii* at different timepoints, compared with the expression level of β-actin in each group.

Genes	*C. auris*			*C. haemulonii*		
	**8 hpi**	**96 hpi**	***P* value#**	**8 hpi**	**96 hpi**	***P* value#**
**Proinflammatory and inflammatory cytokines**						
tnfa	1.57**	1.13^{NS}	0.0077	1.04^{NS}	1.09^{NS}	0.9579
ifng	0.78*	0.40****	0.0007	0.81^{NS}	0.76^{NS}	0.8620
il1b	1.55**	0.70*	0.0003	0.78^{NS}	1.24^{NS}	0.0443
il6	0.88^{NS}	1.47*	0.0130	1.14^{NS}	2.46****	<0.0001
il8	2.98****	0.84^{NS}	<0.0001	1.49**	1.03^{NS}	0.0093
il10	1.48*	1.01^{NS}	0.0242	0.81^{NS}	1.52**	0.0394
il17a	1.11^{NS}	1.97**	0.0019	2.17***	1.24^{NS}	0.0040
**Leukocyte activities**						
inos	0.78^{NS}	1.87***	<0.0001	0.68^{NS}	1.66**	0.0003
mpx	1.09^{NS}	1.22^{NS}	0.4064	0.92^{NS}	3.06****	<0.0001
**Matrix metalloproteinases**						
mmp9	0.39****	1.13^{NS}	<0.0001	1.95****	1.30****	<0.0001
mmp13	1.28^{NS}	1.22^{NS}	0.8342	1.36^{NS}	1.32^{NS}	0.9638
**Inflammatory regulators**						
myd88	1.61*	0.95^{NS}	0.0107	1.11^{NS}	1.10^{NS}	0.9960
nfkb	0.85^{NS}	1.03^{NS}	0.0353	1.29*	1.86***	0.0014
jak2	2.16****	0.37***	<0.0001	0.33***	0.21****	0.0342
stat3	1.56*	1.19^{NS}	0.0003	2.11***	1.49*	<0.0001
**Regulatory T-cells**						
foxp3a	2.02***	0.87^{NS}	0.0002	0.72^{NS}	2.44***	<0.0001
foxp3b	1.25*	0.82^{NS}	0.0016	0.80^{NS}	1.41*	0.0001

Note: The normalized expression levels were analyzed with the Livak method ($2^{-\Delta\Delta CT}$): < 1, downregulation; 1, basal; > 1, upregulation.

Abbreviations and symbols: hpi, hour post infection; Compared gene expression level with β-actin in each group: ^{NS}, not significant; *, $P < 0.5$; **, $P < 0.01$; ***, $P < 0.001$; ****, $P < 0.0001$; #, Comparison of expression level at different timepoint in each group.

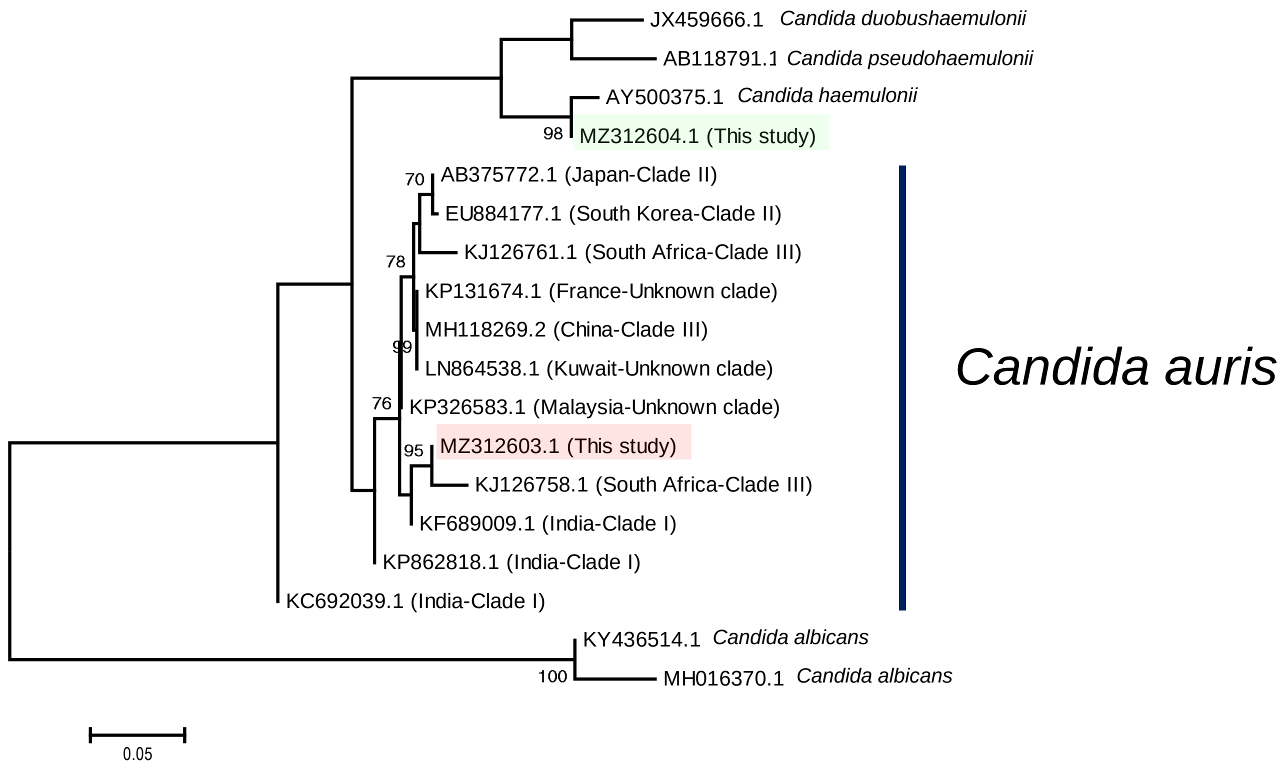

Figure S1. Phylogenic tree using ITS sequences of emerging strains in this study (aligned by ClustalW and drawn by p-distance model and Neighbor-Joining with bootstrap analysis by MEGA program version 6). The information of ITS sequences in this analysis was adapted from [70].

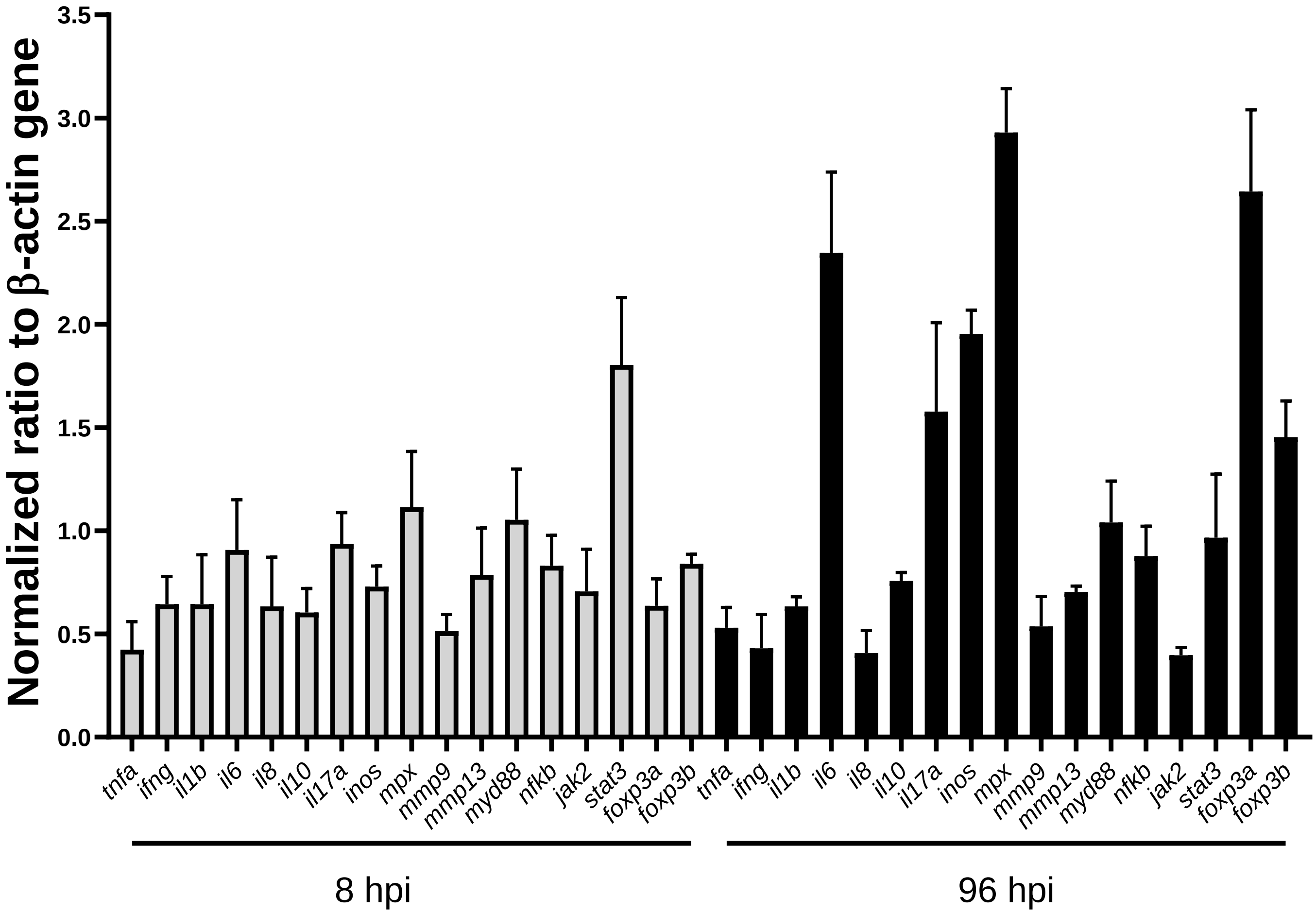

Figure S2. qPCR analysis of the gene expression levels in zebrafish infected with *Candida albicans* ATCC24433 as a control strain in this study. Each experimental group of 20 zebrafish was injected with 10^7 CFU yeast suspension. The normalized ratios of expression were calculated by comparison with the level of expression of the β-actin gene at 8 hpi. Each experiment was performed in triplicate. **Abbreviations:** hpi, hour-post-infection

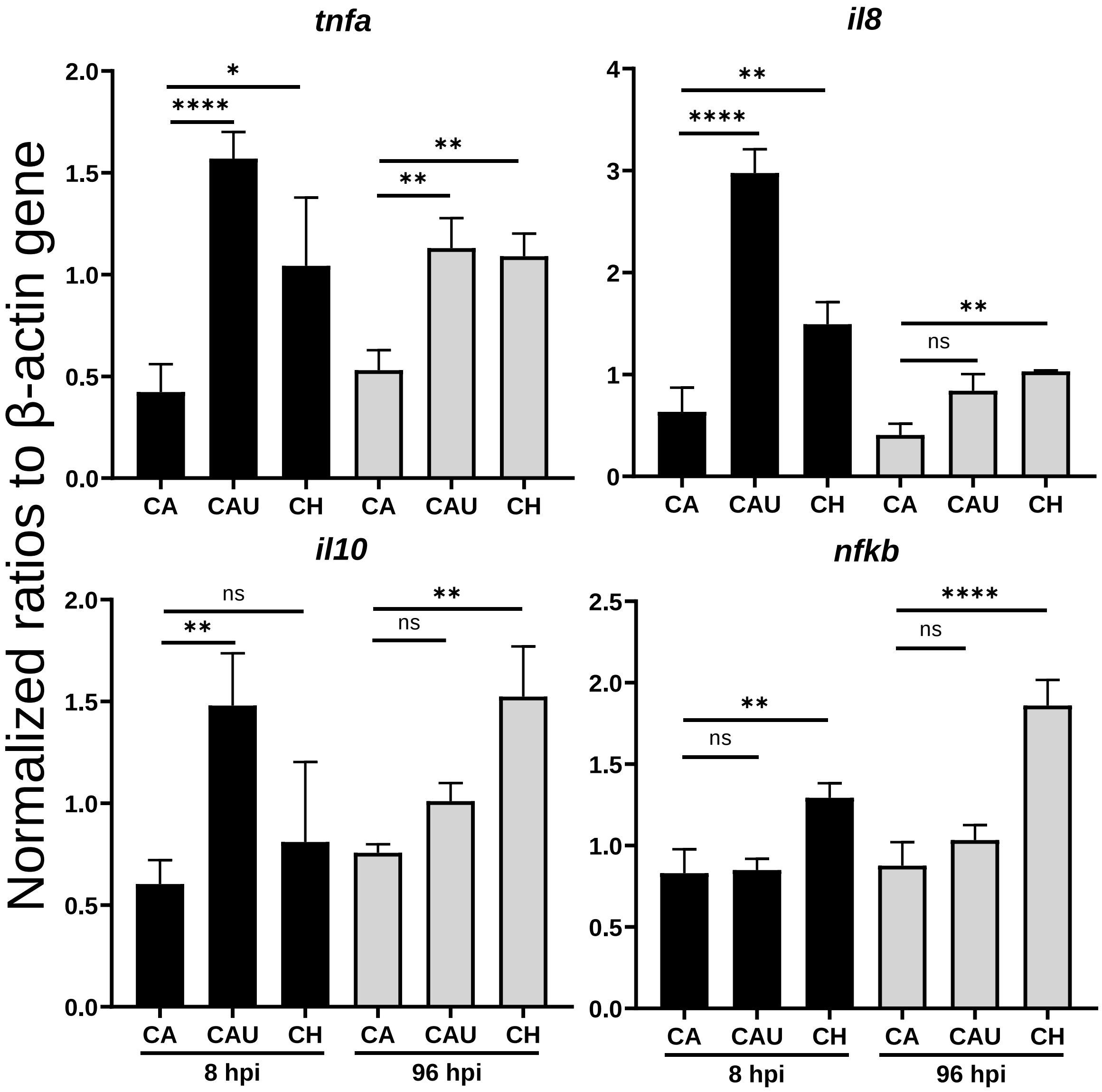

Figure S3. qPCR analysis indicating the high alterations of the expression levels of *tnfa, il8, il10,* and *nfkb* in zebrafish infected with emerging *Candida* strains. Each experimental group of 20 zebrafish was injected with 10^7 CFU cells of one of *C. albicans* ATCC24433 (CA), *C. auris* strain SI-18-CAU-HEM (CAU), or *C. haemulonii* strain SI-21-CH-PLF (CH). The normalized ratios of expression were calculated by comparison with the level of expression of the β-actin gene in each group at 8 hpi. Each experiment was performed in triplicate. **Abbreviations and symbols:** hpi, hour-post-infection; ^{ns}, not significant; *, $P < 0.5$; **, $P < 0.01$; ***, $P < 0.001$; ****, $P < 0.0001$.
